# Gut resistome plasticity in pediatric patients undergoing hematopoietic stem cell transplantation

**DOI:** 10.1038/s41598-019-42222-w

**Published:** 2019-04-04

**Authors:** Federica D’Amico, Matteo Soverini, Daniele Zama, Clarissa Consolandi, Marco Severgnini, Arcangelo Prete, Andrea Pession, Monica Barone, Silvia Turroni, Elena Biagi, Patrizia Brigidi, Riccardo Masetti, Simone Rampelli, Marco Candela

**Affiliations:** 1 Unit of Microbial Ecology of Health, Department of Pharmacy and Biotechnology, University of Bologna, Via Belmeloro 6, Bologna, 40126 Italy; 2 Pediatric Oncology and Hematology Unit “Lalla Seràgnoli”, Department of Pediatrics, University of Bologna, Sant’Orsola Malpighi Hospital, Via Massarenti 11, Bologna, 40138 Italy; 3 Institute of Biomedical Technologies, Italian National Research Council, Milan, Italy

## Abstract

The gut microbiome of pediatric patients undergoing allo-hematopoietic stem cell transplantation (HSCT) has recently been considered as a potential reservoir of antimicrobial resistance, with important implications in terms of patient mortality rate. By means of shotgun metagenomics, here we explored the dynamics of the gut resistome – i.e. the pattern of antibiotic resistance genes provided by the gut microbiome – in eight pediatric patients undergoing HSCT, half of whom developed acute Graft-versus-Host Disease (aGvHD). According to our findings, the patients developing aGvHD are characterized by post-HSCT expansion of their gut resistome, involving the acquisition of new resistances, as well as the consolidation of those already present before HSCT. Interestingly, the aGvHD-associated bloom in resistome diversity is not limited to genes coding for resistance to the antibiotics administered along the therapeutic course, but rather involves a broad pattern of different resistance classes, including multidrug resistance, as well as resistance to macrolides, aminoglycosides, tetracyclines and beta-lactams. Our data stress the relevance of mapping the gut resistome in HSCT pediatric patients to define the most appropriate anti-infective treatment post HSCT.

## Introduction

The rate of infection by antibiotic-resistant bacteria (ARB) is continuously raising worldwide, particularly because of the selective pressure resulting from the increasing usage of broad-spectrum antibiotics^1^. This burden of ARB is of particular relevance for hematological patients, who undergo frequent antimicrobial prophylaxis and treatments^2^. The prolonged exposure to health care settings may indeed favor the progressive accumulation of antimicrobial resistance (AMR) genes in the gut microbiome (GM) of patients^3^. Consequently, opportunistic ARB can accumulate in intestinal niches, where they can take advantage of the chemotherapy-induced damage to the gut epithelium and the overlapping neutropenia, spreading through the gut wall and causing life-threating systemic infections^4,5^. In patients who have received an allogeneic hematopoietic stem cell transplantation (HSCT), systemic infections with ARB have indeed been associated with a non-relapse mortality rate from 36 to 95%^5–7^. Furthermore, gut colonization by ARB and associated systemic infections may strongly influence the process of immune system recovery following HSCT, thus affecting the incidence of acute Graft-versus-Host Disease (aGvHD)^8,9^.

Although the relevance of ARB in HSCT is well recognized, to date the search for ARB in HSCT patients has been exclusively carried out by means of routine culture-dependent tests on rectal swabs^10–12^. Even if this represents a robust diagnostic approach, culture-dependent assessments of antibiotic resistance do not allow the evaluation of the whole gut resistome, defined as the overall pattern of AMR genes and the corresponding ARB present in the GM^13^. The gut resistome has recently been recognized as an important and dynamic reservoir of AMR genes, which can no longer be ignored when assessing antibiotic resistance^14–16^. In fact, it represents a basin of AMR genes that can be transferred to passenger pathogens or opportunistic bacteria by horizontal gene transfer, with serious repercussions on human health^17–19^.

In this scenario, the molecular assessment of the structure, ecology and evolution of the gut resistome in HSCT patients has become of strategic importance, allowing to understand the dynamics that govern the ARB establishment in such patients. Particularly, the gut resistome characterization by shotgun metagenomics has been indicated as a unique and sensitive approach to understanding the genetic and biological effects of AMR in HSCT, and studies in this direction have recently been encouraged^9^.

In our previous research, we provided the 16S rDNA-based phylogenetic description of the GM trajectory in 10 pediatric patients undergoing allo-HSCT^20^. Based on our findings, peculiarities of the structure and temporal dynamics of the GM – such as the presence and abundance of mutualistic short-chain fatty acid producers - are a relevant factor for the success of HSCT. In the present manuscript, we performed a whole-genome shotgun (WGS) metagenome sequencing of the fecal DNA from eight subjects (four developing aGvHD and four aGvHD-negative) from Biagi *et al*.^20^. This work provides – to our knowledge for the first time – some glimpses on the gut resistome structure and its evolutionary trajectory in HSCT pediatric patients, before and after transplantation. According to our findings, the GM of post-HSCT pediatric patients is characterized by the bloom of several broad-spectrum AMR genes, whose diversity and abundance may have implications on the success of anti-microbial therapies.

## Results

### Gut resistome structure in pediatric patients undergoing HSCT and healthy controls

**Figure 1:**
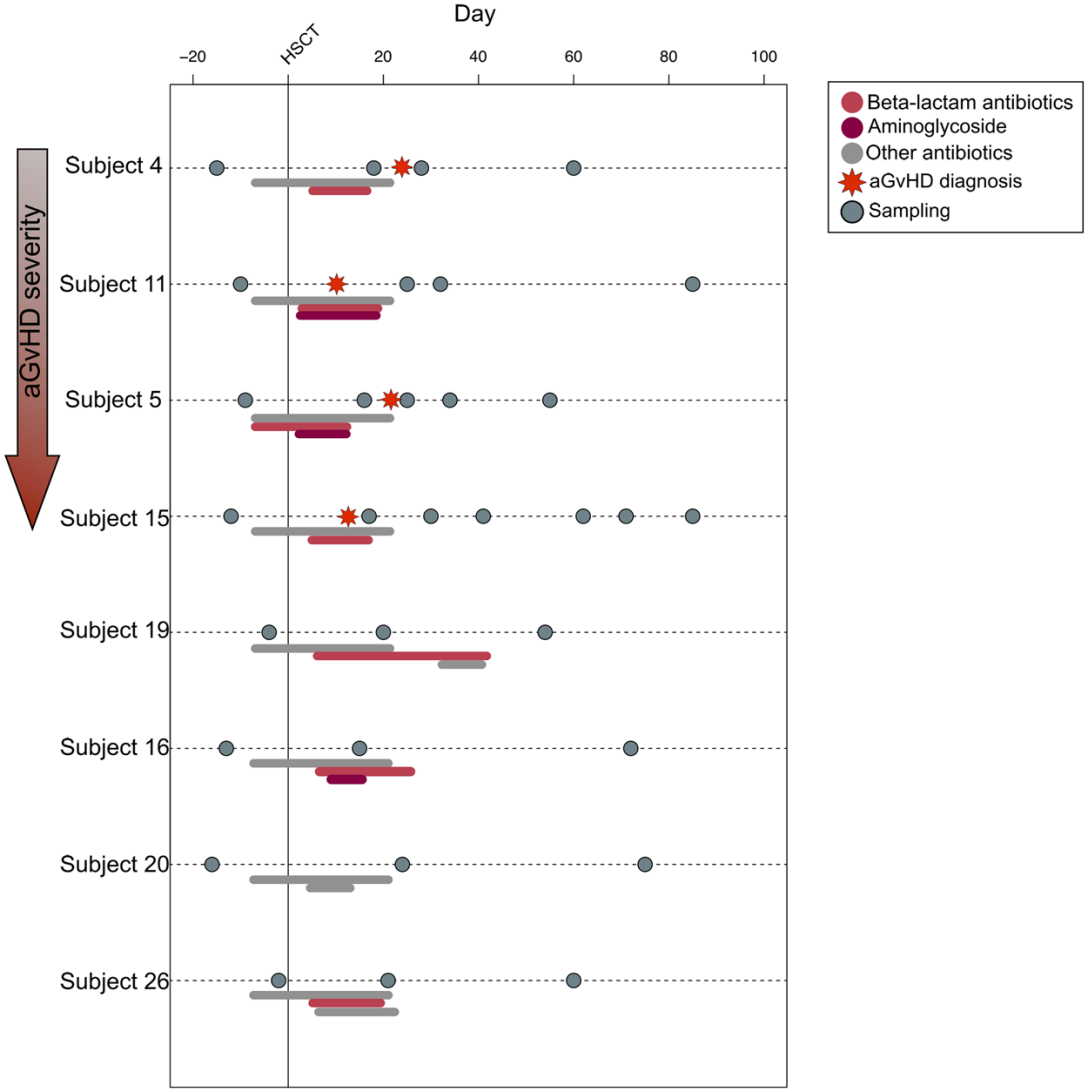
Schematic representation of the sampling time for each enrolled patient. HSCT is represented as a vertical line in the graph, while the occurrence of aGvHD is highlighted with a red star on the subject timeline. The bars below each timeline indicate the individual antimicrobial treatments by antibiotic class (see the legend at the top right).

By means of shotgun metagenomics analysis of fecal DNA, we characterized the resistome configuration in eight pediatric patients undergoing HSCT from Biagi *et al*.^20^. Fecal collection was performed before transplantation and at different time points (at least 3 per subject) post HSCT, for a total of 32 fecal samples (Fig. 1). We generated 66,738,475 high-quality reads (mean ± SEM, 2,085,577 ± 333,987). Reads were analyzed following the same bioinformatic procedure described by Rampelli *et al*.^21^. AMR reads were clustered into 45 ARUs (Antibiotic Resistance Units), each characterized by amino acid sequence identity of at least 30%. The most abundant sequence of each ARU was selected as representative and used for functional assignment.

**Figure 2:**
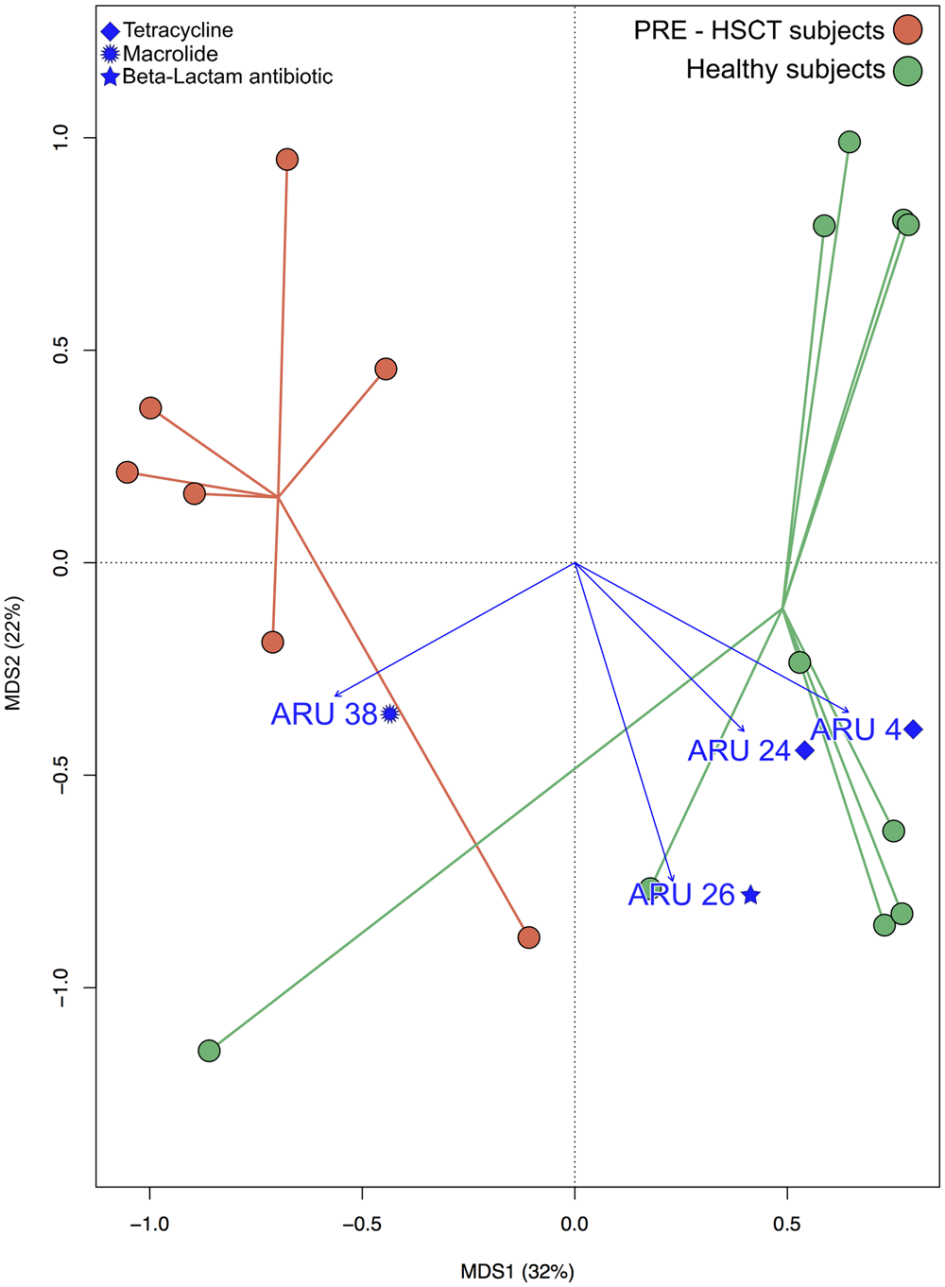
Gut resistome structure of pre-HSCT pediatric patients and healthy subjects. Bray-Curtis distance-based Principal Coordinates Analysis showing separation between the gut resistome of pre-transplant pediatric patients and healthy controls (from Rampelli *et al*.^21^). Permutation test with pseudo-F ratios (Adonis), p = 0.001. Antibiotic Resistance Units (ARUs) with a significant correlation with the bidimensional space are represented with a blue arrow.

In order to highlight peculiarities in pre-HSCT pediatric patients, we compared their resistome configuration with that of 10 healthy Italian adults from Rampelli *et al*.^21^. Bray-Curtis distance-based PCoA shows significant segregation in the gut AMR gene composition between pre-HSCT patients and healthy controls (permutation test with pseudo-F ratios, p = 0.001) (Fig. 2). In particular, the GM of pre-HSCT patients was found to be enriched in AMR genes coding for resistance to macrolides (ARU38). On the other hand, tetracycline (ARUs4 and 24) and beta-lactam (ARU26) AMR genes were found to be more represented in healthy controls. In Supplementary Fig. 1, the relative abundance of each ARU in pre-HSCT patients and healthy controls is provided. For each ARU, the p value of the difference in abundance between patients and controls is also reported.

### Gut resistome dynamics in pediatric patients after HSCT

**Figure 3:**
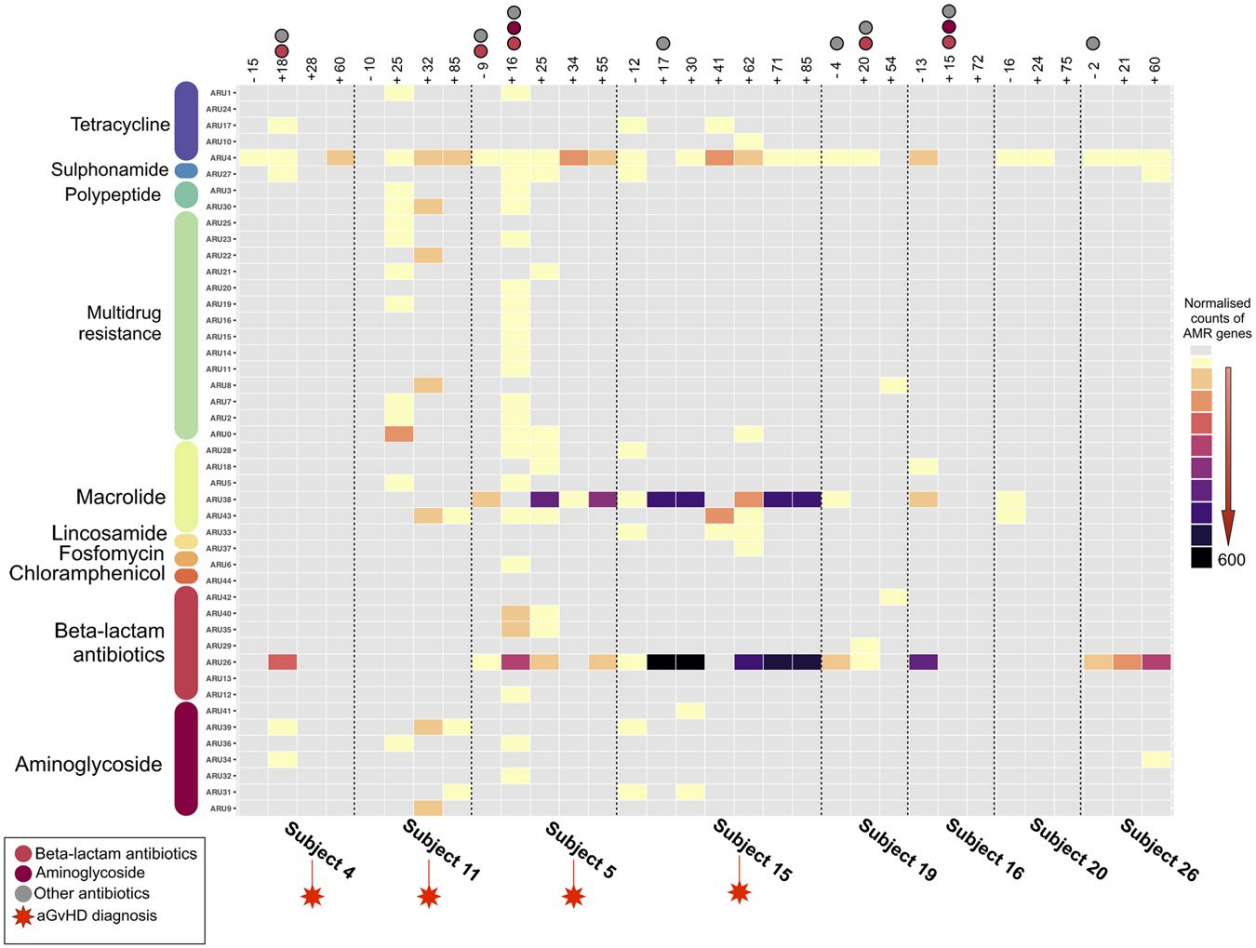
ARUs trajectory over time in pediatric patients undergoing HSCT. Rectangles indicate the distribution of ARU abundances across time points for each subject, normalized by AMR gene count and represented with different colors, from gray (0 count) to black (600 counts). A black dotted vertical line is used to separate the sample sets of patients. aGvHD-positive subjects are highlighted with a red star. The presence of colored circles indicates the antibiotic intake during the specific time point (light red for beta-lactam antibiotics, dark red for aminoglycosides and gray for other antibiotics). Antibiotic Resistance Units (ARUs) are grouped by class of antibiotic, based on the assigned protein function.

We analyzed the temporal dynamics of the gut resistome in the pediatric patients undergoing HSCT, by building a heat map showing the ARUs abundance in all pre- and post-transplant samples collected (Fig. 3). During the sampling period, four patients developed aGvHD with different grade of severity (from I to IV, Supplementary Table 1). According to our findings, most of the detected AMR genes code for multidrug resistance as well as resistance to tetracyclines, macrolides, beta-lactams and aminoglycosides. Interestingly, patients developing aGvHD show a peculiar resistome trajectory, being enriched in AMR genes compared to non-aGvHD patients. In particular, aGvHD patients show higher abundance and diversity of genes belonging to multidrug, macrolide and aminoglycoside resistance classes. All these resistance classes are represented by multiple ARUs, all of which seem to be acquired post-HSCT. For 3 out of 4 aGvHD patients (n. 4, 11 and 5) we observed a bloom of AMR genes after HSCT. Interestingly, this rise in antibiotic resistance was attributable to multiple bacterial species and thus the resistance acquisition occurred at the ecosystem level. In particular, according to our findings, for patients n. 4, 11 and 5, AMR genes were attributed to 5, 6 and 11 different microbial species, respectively. With regard to single AMR genes, we observed an increasing trend for ARU4 – present in almost all pre-HSCT samples and coding for a tetracycline inhibitor - in aGvHD-positive patients compared to non-aGvHD patients, in whom the abundance of this ARU remains overall stable during the sampling period. It is worth noting that subjects 5 and 15, developing grade III and IV aGvHD, show a high abundance of two ARUs, belonging to different AMR classes: ARU26, assigned to beta-lactamase CFXA3 and ARU38, coding for resistance to erythromycin, belonging to the macrolide resistance group. As observed for ARU4, both of these resistance genes were detected in pre-HSCT samples and found to persist with higher gene counts up to more than two months after transplant.

### Microbial ecology of the AMR classes in HSCT pediatric patients

**Figure 4:**
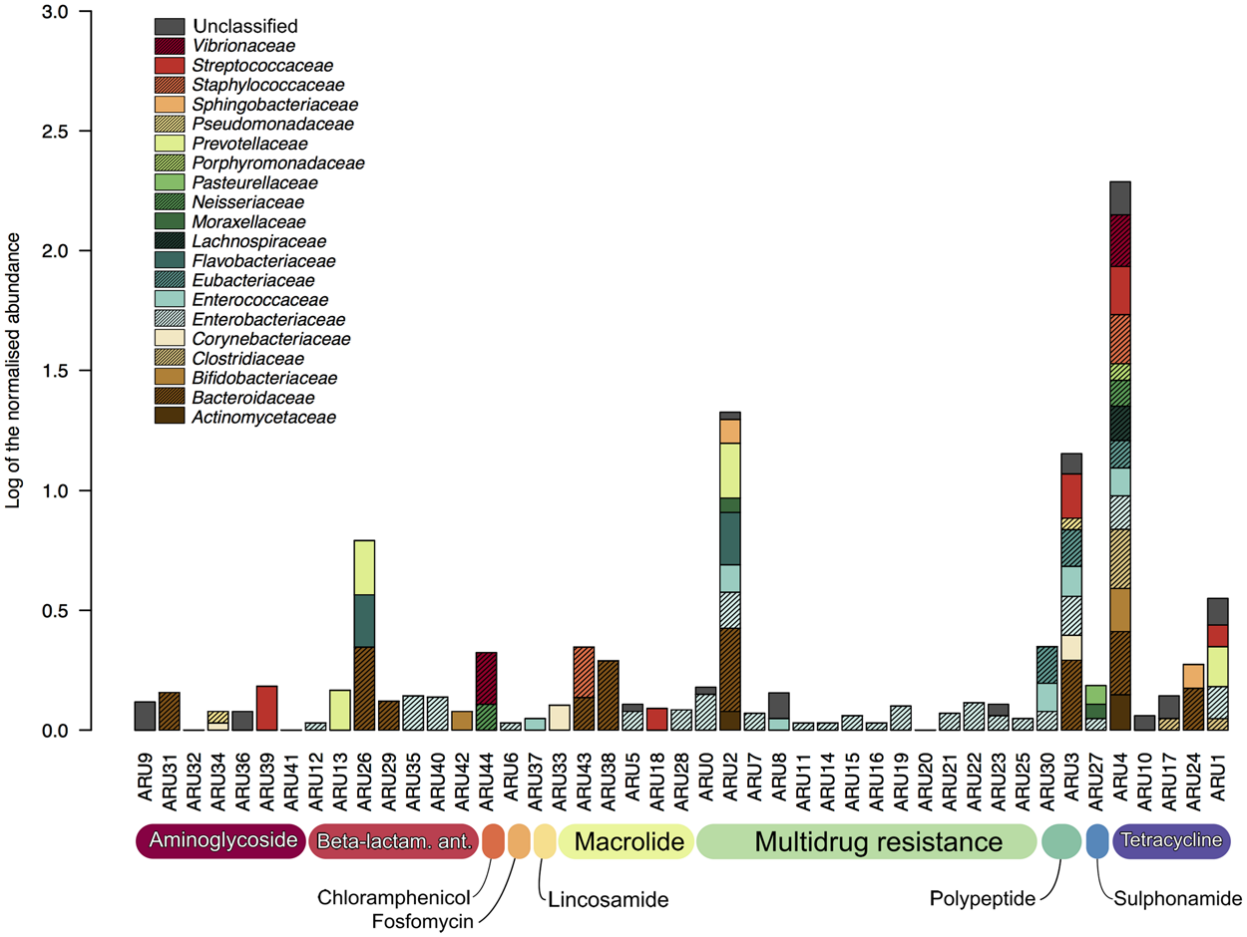
Microbial ecology of Antibiotic Resistance Units (ARUs). For each ARU, total abundance and family-level distribution are represented. ARUs are grouped by class of antibiotic, based on the assigned protein function. The reported values were normalized using a logarithmic scale. Ant., antibiotics.

In order to explore the taxonomy of the gut resistome, each of the 45 previously detected ARUs was assigned at the family level (Fig. 4). Our results highlight a wide distribution of AMR genes at this taxonomic level, including both environmental and intestinal microorganisms. Particularly, when focusing on AMR genes characterizing aGvHD patients, we observed that those acquired post-HSCT (i.e. those coding for multidrug, macrolide and aminoglycoside resistances) were assigned to bacterial families of putative intestinal origin, such as *Bacteroidaceae*, *Enterobacteriaceae*, *Enterococcaceae*, *Eubacteriaceae* and *Streptococcaceae*^22^, as well as to more environmental and cosmopolitan microorganisms, such as *Pseudomonadaceae* and *Sphingobacteriaceae*^23^. Conversely, for what concerns the ARUs that were already present in pre-HSCT samples - and whose abundance was found to increase post-HSCT – i.e. ARU4 (tetracycline inhibitor), ARU26 (β-lactamase CFXA3) and ARU38 (erythromycin resistance), a prevalent intestinal origin was determined. In particular, ARU4 is common to several GM components, including *Bifidobacteriaceae*, *Bacteroidaceae*, *Clostridiaceae*, *Enterobacteriaceae*, *Enterococcaceae*, *Eubacteriaceae*, *Lachnospiraceae* and *Streptococcaceae*, among others. Differently, ARU26 and ARU38 show a limited taxonomic diversity, with the first being assigned to three intestinal groups, *Bacteroidaceae*, *Eubacteriaceae* and *Prevotellaceae*, and the second only to *Bacteroidaceae*. These last ARUs, which characterize higher grade aGvHD, were also assigned at the species level. Particularly, the ARU26 was assigned to *Bacteroides* sp. *D1*, *Prevotella intermedia*, *Capnocytophaga ochracea* and *Bacteroides fragilis* species*;* conversely the ARU38 was assigned to *B. fragilis* and *Bacteroides* sp. (Supplementary Fig. 2).

## Discussion

In order to highlight the impact of previous therapeutic treatments on the gut resistome of HSCT pediatric patients, we firstly compared their pre-HSCT gut resistome configuration with the AMR gene composition of 10 healthy Italian subjects from Rampelli *et al*.^21^. According to our findings, pre-transplant pediatric patients possess an overall gut resistome structure different from that of healthy individuals, possibly shaped by the previous prolonged exposure to health care settings and being enriched in AMR genes providing for macrolide resistance^17,18^. However, it should be stressed that the comparison of the AMR composition was performed between children and adults, and therefore the data need to be taken with adequate caution as the gut microbiome structure is known to change with age. Afterward, we focused the analysis on temporal variations of the gut resistome in the pediatric patients undergoing HSCT. Interestingly, our data highlight a distinctive gut resistome trajectory in patients developing aGvHD, involving not only the consolidation of AMR genes already present before transplanting, but also the acquisition of a vast number of new AMR genes following HSCT. Coding for multidrug, macrolide and aminoglycoside resistance classes, these newly acquired AMR genes were assigned to different bacterial families, including microorganisms of intestinal origin as *Bacteroidaceae*, *Enterobacteriaceae*, *Enterococcaceae*, *Eubacteriaceae* and *Streptococcaceae*^22^, as well as cosmopolitan bacteria, such as *Pseudomonadaceae* and *Sphingobacteriaceae*^23^. The gut resistome of aGvHD-positive patients was also found to be characterized by the increase in abundance of AMR genes already present before HSCT. In particular, a consistent post-HSCT bloom was detected only in aGvHD cases for AMR genes coding for a tetracycline inhibitor, β-lactamase CFXA3 and erythromycin resistance. Interestingly, these AMR genes were assigned to GM components, including *Bacteroidaceae*, *Prevotellaceae*, *Lachnospiraceae* and *Streptococcaceae*. It is also worth noting that the bloom of β-lactamase CFXA3 and erythromycin resistance – prevalently attributed to the major GM species of *Bacteroides* sp. and *B. fragilis*– was found to be associated with higher aGvHD severity (grade III and IV).

In conclusion, our assessment of the gut resistome dynamics in eight pediatric patients undergoing HSCT allowed – to our knowledge for the first time - to shed some light on the microbial ecology of ARB in HSCT, going beyond the limit of the traditional culture-dependent studies. Despite the low number of subjects, we indeed provided evidence that aGvHD onset is associated with a peculiar trajectory of the personal gut resistome following HSCT. Even if all patients received fluoroquinolone antibiotic prophylaxis from day −9 to day 21 and anti-infective therapy based on beta-lactam antibiotics after transplant, only aGvHD subjects showed an extremely diversified and rich gut resistome, with a pattern of AMR genes far exceeding the selective pressure due to the administered antibiotics. In particular, after HSCT, the resistome of pediatric patients developing aGvHD acquires a new and diversified pattern of AMR genes, either from enteric and environmental microorganisms, and including multidrug resistance, as well as resistances to macrolide and aminoglycoside antibiotic classes. However, in parallel with the acquisition of new AMR genes, the aGvHD development is also associated with a bloom of internal AMR genes, already present in the individual gut resistome before the HSCT, and provided by major gut microbiome components such as *Bacteroides* sp. Particularly, this last element leads to the consolidation of AMR genes such as tetracycline inhibitor, β-lactamase CFXA3 and erythromycin resistances, the latter two associated with a high aGvHD severity grade. Taken together, our research indicate that the individual GM of HSCT patients can thus act as a dynamic reservoir of ARB, with the potential to implement the AMR gene pattern following HSCT. According to our findings, this aGvHD-associated magnification process of the individual gut resistome involve variations in the abundance of endogenous gut microbiome ARB, as well as the acquisition of allochthonous ARB, of enteric or environmental nature. Even if these data must be confirmed on a larger cohort, in a recently published research^24^, it has been assessed the gut resistome dynamics in 12 subjects exposed to an antibiotic therapy. Results highlighted a plastic resistome response which partially resembled our observations. Indeed, according to the authors, four days post treatment it was observed an enrichment of AMR genes, not limited to the ones targeted to the administered antibiotics. The inherently plastic behavior of the human gut resistome supports the importance of WGS-based resistome surveys in pediatric HSCT patients, allowing a better comprehension of the ecological dynamics of antibiotic resistance in aGvHD-positive cases, with the final goal of allowing a better refinement of antibacterial therapies.

## Methods

### Sample collection

This study used genomic DNA extracted from fecal samples of eight pediatric patients (mean age, 9.9 years) from Biagi *et al*.^20^, who underwent allo-HSCT for high risk acute leukemia. Four patients out of the eight developed moderate (I-II grade) to severe (III-IV stage) aGvHD (Supplementary Table 1). Fecal samples were collected before HSCT and at different time points after the transplant, up to about 85 days post-HSCT, for a total of 32 samples (Fig. 1). Sample collection, storage and DNA extraction procedures are fully reported in Biagi *et al*.^20^. Because of episodes of febrile neutropenia occurred after the chemotherapy, patients received an empirical treatment based on a third-generation cephalosporin with activity against *Pseudomonas* before HSCT. Informed consent was obtained for all the subjects enrolled by parents and/or legal guardians. The study was approved by the Ethics Committee of the Sant’Orsola-Malpighi Hospital-University of Bologna (ref. number 19/2013/U/Tess). All methods were performed in accordance with the relevant guidelines and regulations.

Publicly available shotgun metagenomic sequencing data from fecal samples of 10 healthy Italian subjects (mean age, 31.9 years) from Rampelli *et al*.^21^ (MG-RAST: http://metagenomics.anl.gov/linkin.cgi?project=8810) were retrieved for comparative purposes and processed as described below.

### Whole-genome shotgun (WSG) sequencing

DNA libraries were prepared using Illumina TruSeq DNA PCR-Free Low throughput Library Preparation Kit, following the manufacturer’s instructions with few modifications. 100 nanograms of each genomic DNA was fragmented using a Covaris S2 sonication device in order to obtain 350 bp inserts. After sonication, DNA fragments were end-repaired and the appropriate library size was selected using different ratios of the Sample Purification Beads, then the samples were A-tailed and ligated to adapters. Because of the low qualities of the library obtained, an “Enrich DNA Fragments” step was introduced according to the manufactures instructions (Illumina TruSeq DNA Sample Preparation Kit). Finally, size and concentration of DNA fragments were assessed using KAPA Library Quantification Kits (Kapa Biosystems) specific for the Illumina libraries. Approximately 8 pM libraries were paired-end sequenced (2 × 100 bp) on Illumina GAIIx platform: three samples were simultaneously sequenced in each line.

### Gut resistome analysis

Functional annotation of the sequences was performed as previously described in Rampelli *et al*.^21^. Briefly, shotgun reads were quality filtered using the human sequence removal pipeline from the Human Microbiome Project^25^, and filtered reads were assembled in contigs using the MetaVelvet tool^26^. Raw sequence reads were deposited in the National Center for Biotechnology Information Sequence Read Archive (https://www.ncbi.nlm.nih.gov/bioproject/PRJNA525982). Protein sequences from the Antibiotic Resistance Genes Database (ARDB)^27^ were screened against the assembled metagenomes using TBLASTN^28^. Only alignments with identity ≥80% and alignment length of at least 200 residues were retained for further analysis. When multiple hits were present, the best one was selected according to three criteria with the following priority: (i) percentage of identity and length of the alignment, (ii) function showing the highest number of hit, and (iii) presence of the corresponding microorganism in the respective gut ecosystem^20,29^. For further analysis, the target resistance genes were normalized using the number of reads in the corresponding sample. Taxonomic classification of the identified sequences was retrieved from the results of TBLASTN^28^. The amino acid sequences of the select proteins were clustered into Antibiotic Resistance Units (ARUs) at 30% identity level using UCLUST^30^ (Supplementary Table 2). The most abundant sequence of each ARU was selected as a representative sequence and re-classified using BLASTP^28^ and ARDB^27^. ARU table containing resistance abundance across the samples was built using the script “make_otu_table.py” in QIIME^31^ and used for further analysis as described below.

### Bioinformatics and statistical analysis

The ARU table was used as input for a Principal Coordinates Analysis (PCoA) based on Bray-Curtis distances between samples. PCoA graphs were generated using the “vegan” package (http://www.cran.r-project.org/package=vegan) in R studio version 1.0.153, and data separation was tested by permutation test with pseudo-F ratios (function “Adonis” in “vegan”). The ARU table was also used to build a heat map of the normalized ARU abundances before and after transplantation for all patients (“ggplot2” package^32^). ARUs were superimposed on the bidimensional space using the function “envfit” of the “vegan” package and only AMR genes showing a significant correlation were plotted. Significant differences in ARU table between pre-HSCT patients and healthy controls were assessed by Wilcoxon signed rank-sum test. False discovery rate (FDR) < 0.05 was considered as statistically significant.

## Supplementary information

Supplementary Information
