# Supplementary material for: Gut resistome plasticity in pediatric patients undergoing hematopoietic stem cell transplantation
Source: Sci Rep. 2019 Apr 4;9:5649. doi: 10.1038/s41598-019-42222-w (PMC6449395; doi:10.1038/s41598-019-42222-w)
Supplement: Supplementary file 1 — Supplementary Information [file 41598_2019_42222_MOESM1_ESM.pdf]

Gut resistome plasticity in pediatric patients undergoing hematopoietic stem cell transplantation

Federica D'Amico, Matteo Soverini, Daniele Zama, Clarissa Consolandi, Marco Severgnini, Arcangelo Prete, Andrea Pession, Monica Barone, Silvia Turroni, Elena Biagi, Patrizia Brigidi, Riccardo Masetti, Simone Rampelli and Marco Candela.

	pre-HSCT	pre-HSCT SD	Healthy subjects	Healthy subjects SD	p value
ARU0	0.000	0.000	0.000	0.000	NA
ARU1	0.000	0.000	0.000	0.000	NA
ARU2	0.000	0.000	0.000	0.000	NA
ARU3	0.000	0.000	0.000	0.000	NA
ARU4	7.714	5.936	72.800	66.530	0.008
ARU5	0.000	0.000	0.000	0.000	NA
ARU6	0.000	0.000	0.000	0.000	NA
ARU7	0.000	0.000	0.000	0.000	NA
ARU8	0.000	0.000	0.000	0.000	NA
ARU9	0.000	0.000	0.000	0.000	NA
ARU10	0.000	0.000	0.000	0.000	NA
ARU11	0.000	0.000	0.000	0.000	NA
ARU12	0.000	0.000	0.000	0.000	NA
ARU13	0.000	0.000	4.600	9.709	0.257
ARU14	0.000	0.000	0.000	0.000	NA
ARU15	0.000	0.000	0.000	0.000	NA
ARU16	0.000	0.000	0.000	0.000	NA
ARU17	0.143	0.378	0.500	1.581	0.931
ARU18	0.714	1.890	0.000	0.000	0.282
ARU19	0.000	0.000	0.000	0.000	NA
ARU20	0.000	0.000	0.000	0.000	NA
ARU21	0.000	0.000	0.000	0.000	NA
ARU22	0.000	0.000	0.000	0.000	NA
ARU23	0.000	0.000	0.000	0.000	NA
ARU24	0.000	0.000	6.500	11.257	0.076
ARU25	0.000	0.000	0.000	0.000	NA
ARU26	18.857	36.334	25.400	26.056	0.654
ARU27	0.143	0.378	0.000	0.000	0.282
ARU28	0.143	0.378	0.000	0.000	0.282
ARU29	0.000	0.000	0.700	1.636	0.257
ARU30	0.000	0.000	3.400	7.230	0.257
ARU31	0.286	0.756	2.600	5.797	0.714
ARU32	0.000	0.000	0.000	0.000	NA
ARU33	0.143	0.378	0.000	0.000	0.282
ARU34	0.000	0.000	0.000	0.000	NA
ARU35	0.000	0.000	0.000	0.000	NA
ARU36	0.000	0.000	0.000	0.000	NA
ARU37	0.000	0.000	0.000	0.000	NA
ARU38	8.000	8.185	2.900	7.578	0.071
ARU39	0.143	0.378	4.600	6.398	0.203
ARU40	0.000	0.000	0.000	0.000	NA
ARU41	0.000	0.000	0.000	0.000	NA
ARU42	0.000	0.000	0.000	0.000	NA
ARU43	0.286	0.756	8.800	21.953	0.394
ARU44	0.000	0.000	15.500	36.189	0.077

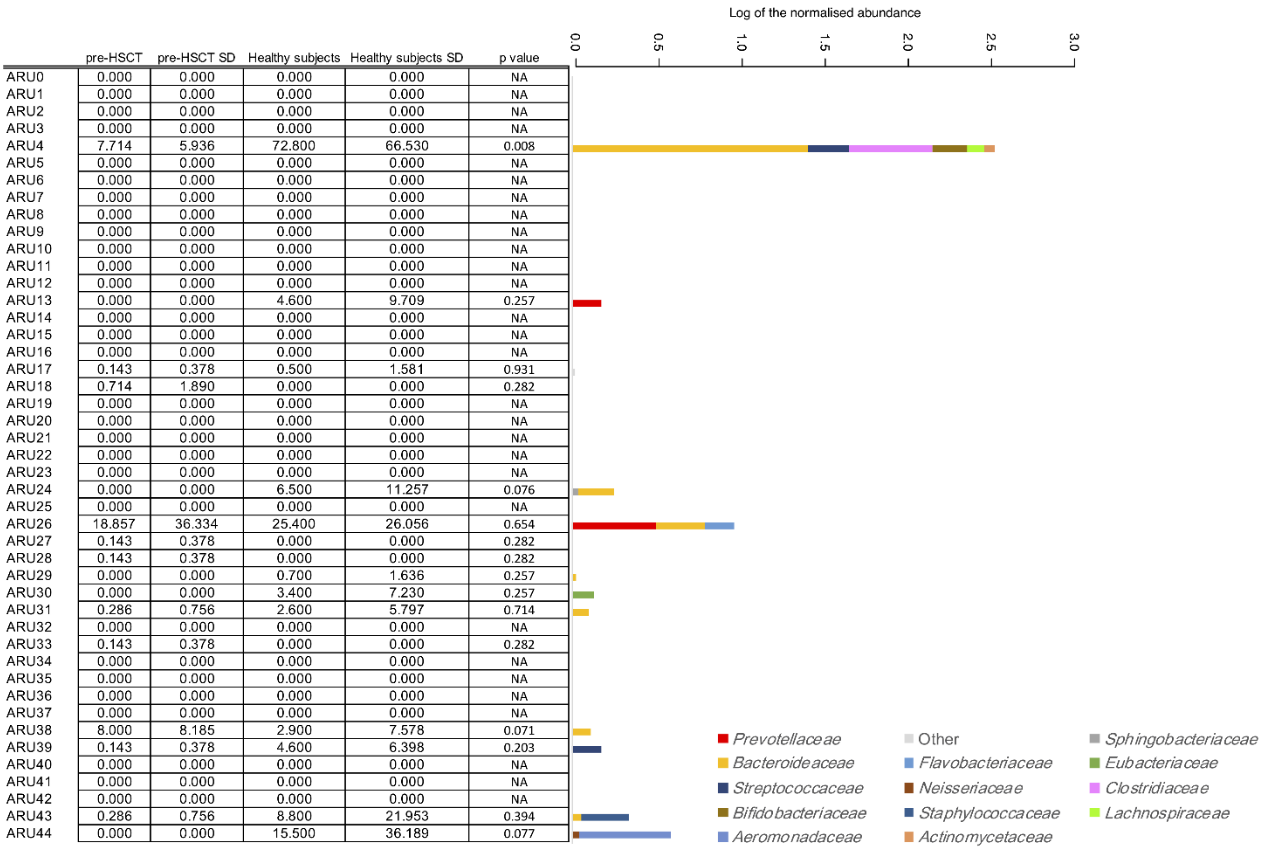

Supplementary Figure 1. Table showing the mean and standard deviation of the relative abundance of each ARU in the two groups analyzed: pre-HSCT samples and healthy subjects. Wilcoxon signed rank-sum test was used to assess significant difference between the two groups of subjects. Bar plots showing the normalized abundance of the families assigned to each ARU for healthy subjects are shown on the right.

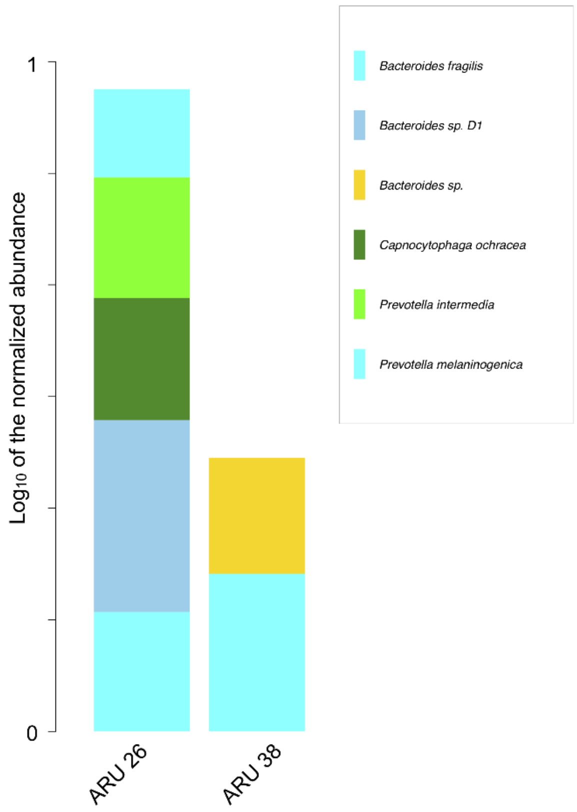

Supplementary Figure 2. Taxonomic classification to species level of ARU26 and ARU38.

Supplementary Table 1. Anagraphical and clinical information of the enrolled patients.

Subject	*Sex/Age*	*Diagnosis*	*Donor Type*	*Stem cell source*	*Conditioning regimen*	*aGvHD prophylaxis*	*TNC /Kg*	*Engraftment (day)*			*aGvHD*			*Outcome*	*Antibiotic prophylaxis*	*Antibiotic treatment*
								PMN	*PLT*	*Grade*	*Site*	*Day*	*Therapy*			
4	F/12	AML	MUD	BM	BU+EDX+ L-PAM	ATG-CSA-MTX	4.2x 10^8	+13	+20	I	Skin	+25	Steroid	ANED 2 years	Levofloxacin ± (-9/+21)	Ceftazidime* (+7/+13) Piperacillin/ tazobactam* (+13/+19)
11	M/9	ALL	MUD	BM	BU+TT+EDX	ATG-CSA-MTX	3.4x 10^8	+15	+27	II	Skin	+11	Steroid	Dead from relapse	Levofloxacin ± (-9+21)	Ceftazidime* (+7/+19) Amikacin# (+7/+21)
5	M/10	ALL	MFD	BM	BU+TT+EDX	CSA	2.9x 10^8	+12	+17	III	Skin	+23	Steroid-PHEC	ANED 2 years +1/12	Levofloxacin ± (-9/+21)	Ceftazidime* (-9/+7) Piperacillin/ tazobactam* (+7/+16) Amikacin# (+3/+16)
15	M/8	ALL	MFD	BM	BU+TT+EDX	CSA	5.8x 10^8	+12	+17	IV	Skin, GI	+11	Steroid-PHEC Infliximab	ANED 1 year +6/12	Levofloxacin ± (-9/+21)	Ceftazidime* (+8/+15)
19	F/10	ALL	MUD	BM	BU+TT+EDX	ATG-CSA-MTX	7.1x 10^8	+34	+35					ANED 1 year +3/12	Levofloxacin ± (-9/+21)	Ceftazidime* (+9/+14) Amikacin# (+11/+18) Meropenem* (+14/+23, +34/+43) Linezolid ± (+34/+41)
16	M/16	ALL	MFD	BM	BU+TT+EDX	CSA	4.6x 10^8	+11	+30					ANED 1 year +6/12	Levofloxacin ± (-9/+21)	Ceftazidime* (+7/+16) Piperacillin/ tazobactam* (+16/+23) Amikacin# (+9/+15)

20	M/7	ALL	MUD	BM	BU+TT+EDX	ATG-CSA-MTX	4.8x 10^8	+12	+15		ANED 1 year +4/12	Levofloxacin ± (-9/+21)	Teicoplanin ± (+9/+16)
26	M/8	AML	MUD	BM	BU+EDX+L-PAM	ATG-CSA-MTX	7.2x 10^8	+12	+21		ANED 1 year +9/12	Levofloxacin ± (-9/+21)	Ceftazidime* (+6/+19) Teicoplanin ± (+10/+21)

Abbrevations: aGvHD=acute graft-versus-host disease; ALL=acute lymphoblastic leukemia; AML=acute myeloid leukemia; ANED=alive no evidence of disease; ATG=anti-thymocyte; BM=bone marrow; BU=busulfan; CSA=cyclosporin A; EDX=cyclophosphamide; F=female; GI=gastrointestinal tract; L-PAM=melphalan; M=male; MFD=match family donor; MTX= methotrexate; MUD=match unrelated donor; PHEC=extracorporeal photopheresis; PMN=polymorphonuclear neutrophil; PLT=platelets; TNC= total nucleated cells; TT=thiotepa; globulin.

*Beta-lactam antibiotic; # Aminoglycoside antibiotic; ± Others. The numbers between parenthesis indicate the starting and the ending day for the antibiotic treatment post-transplant.

Supplementary Table 2. Classification of the Antibiotic Resistance Units (ARUs) detected in the gut resistome of pediatric patients undergoing HSCT and healthy patients. For each ARU, antibiotic class, identity name, resistance type and function are indicated.

Antibiotic Resistance Unit	Antibiotic Class	Identity Name	Resistance Type	Function
[ARU0]	Multidrug resistance	ZP_02346647	acrb	Multidrug resistance efflux pump
[ARU1]	Tetracycline	XP_002333050	tetc	Tetracycline efflux pump
[ARU2]	Multidrug resistance	YP_001460869	mdto	Multidrug resistance efflux pump
[ARU3]	Polypeptide	AAL23678	arna	Resistance to polymyxin
[ARU4]	Tetracycline	BAD46890	tetq	Inhibition of tetracycline
[ARU5]	Macrolide	BAB64542	macb	Macrolide-specific efflux pump
[ARU6]	Fosfidomycin	YP_001477377	rosb	Efflux pump/potassium antiporter system
[ARU7]	Multidrug resistance	YP_002807373	tolc	Multidrug resistance efflux pump
[ARU8]	Multidrug resistance	ZP_04437889	lsa	ABC efflux family that is resistant to MLS antibiotics
[ARU9]	Aminoglycoside	BAG12278	aac6ie	Aminoglycoside N-acetyltransferase
[ARU10]	Tetracycline	P13924	tetl	Tetracycline efflux pump
[ARU11]	Multidrug resistance	YP_403489	mdtk	Multidrug resistance efflux pump
[ARU12]	Beta-lactam antibiotics	ZP_04534300	bl1_ec	Class C beta-lactamase
[ARU13]	Beta-lactam antibiotics	ZP_03615711	tet37	Inhibition of tetracycline
[ARU14]	Multidrug resistance	ABE06672	mdth	Multidrug resistance efflux pump
[ARU15]	Multidrug resistance	BAG80136	mdtm	Multidrug resistance efflux pump
[ARU16]	Multidrug resistance	BAA35851	mdtg	Multidrug resistance efflux pump
[ARU17]	Tetracycline	ACI02010	tet40	Tetracycline efflux pump
[ARU18]	Macrolide	AAQ62546	mefa	Macrolide efflux pump
[ARU19]	Multidrug resistance	YP_002396538	acra	Multidrug resistance efflux pump
[ARU20]	Multidrug resistance	CAS09877	bcr	Multidrug efflux system
[ARU21]	Multidrug resistance	YP_002393667	emrd	Multidrug resistance efflux pump
[ARU22]	Multidrug resistance	ZP_04439153	emea	Multidrug resistance efflux pump

[ARU23]	Multidrug resistance	ZP_03063399	mdtl	Multidrug resistance efflux pump
[ARU24]	Tetracycline	CAC47932	tetx	Tetracycline modification
[ARU25]	Multidrug resistance	YP_410370	mdtn	Multidrug resistance efflux pump
[ARU26]	Beta-lactam antibiotics	ZP_04543532	bl2e_cfxa	Class A beta-lactamase
[ARU27]	Sulphonamide	YP_001969930	sul2	Sulfonamide-resistant dihydropteroate synthase
[ARU28]	Macrolide	AAG14406	mpha	Macrolide phosphotransferase
[ARU29]	Beta-lactam antibiotics	ZP_02068298	bl2e_cepa	Class A beta-lactamase
[ARU30]	Polypeptide	YP_002937728	baca	Undecaprenyl pyrophosphate phosphatase
[ARU31]	Aminoglycoside	ABP57330	ant6ia	Aminoglycoside O-nucleotidylyltransferase
[ARU32]	Aminoglycoside	ACI02996	aac3iia	Aminoglycoside N-acetyltransferase
[ARU33]	Macrolide	ZP_03973325	ermx	Resistance to erythromycin
[ARU34]	Aminoglycoside	CAZ48657	aph6id	Aminoglycoside O-phosphotransferase
[ARU35]	Beta-lactam antibiotics	ACO55744	bl2d_oxa1	Class D beta-lactamase
[ARU36]	Aminoglycoside	BAE76038	ksga	Kasugamycin resistance
[ARU37]	Lincosamide	AAL05554	lnub	Lincosamide nucleotidyltransferase
[ARU38]	Macrolide	AAA27431	ermf	Resistance to erythromycin
[ARU39]	Aminoglycoside	CAP17165	aph3iiia	Aminoglycoside O-phosphotransferase
[ARU40]	Beta-lactam antibiotics	ACI47262	bl2be_ctxm	Class A beta-lactamase
[ARU41]	Aminoglycoside	CAQ06465	aadd	Aminoglycoside O-nucleotidylyltransferase
[ARU42]	Beta-lactam antibiotics	CAH41960	bl3_ccra	Class B beta-lactamase
[ARU43]	Macrolide	BAH18720	ermb	Resistance to erythromycin
[ARU44]	Cloramphenicol	YP_001144149	cata13	Group A chloramphenicol acetyltransferase
